# Revision of the Australian Ceratocanthinae (Coleoptera, Scarabaeoidea, Hybosoridae)

**DOI:** 10.3897/zookeys.339.6033

**Published:** 2013-10-03

**Authors:** Alberto Ballerio

**Affiliations:** 1 Viale Venezia 45, 25123 Brescia, Italy

**Keywords:** Queensland, *Pterorthochaetes*, *Cyphopisthes*, *Mastotermes*, taxonomy, identification key

## Abstract

The Australian fauna of Ceratocanthinae (Coleoptera, Scarabaeoidea, Hybosoridae) is revised. Two genera are present, both shared with Asia, with a total of seven species, all localized in eastern Queensland and all except one, endemic to Australia. *Cyphopisthes* is comprised of three species, two of them new (*Cyphopisthes yorkensis* **sp. n.** and *Cyphopisthes monteithi* **sp. n.**, the latter, together with *Cyphopisthes descarpentriesi* Paulian, 1977 displaying an unusual ecology, with occurrence in the southern Queensland dry rainforest/scrub habitats), and *Pterorthochaetes* is comprised of four species, two of them new (*Pterorthochaetes danielsi* **sp. n.** and *Pterorthochaeres storeyi* **sp. n.**). Descriptions, distribution, ecological remarks and a key to species are provided.

## Introduction

Australia has a very small fauna of Ceratocanthinae (Coleoptera, Scarabaeoidea, Hybosoridae). Only two genera have been recorded thus far: *Cyphopisthes* Gestro, 1899 and *Pterorthochaetes* Gestro, 1899, both genera being mainly distributed in the Oriental region, extending eastwards to the Australasian region, where *Pterorthochaetes* reaches Vanuatu and *Cyphopisthes* reaches New Guinea. In Australia both genera are restricted to eastern Queensland.

The first species described from Australia was the supposedly endemic *Pterorthochaetes simplex* Gestro, 1899. Almost eighty years passed before the description of a second endemic species: *Cyphopisthes descarpentriesi* Paulian, 1977, was published in a revision of the Australian fauna, where a third species, the New Guinean *Pterorthochaetes cribricollis* Gestro, 1899, wasrecorded from Australia and a key to genera and species was provided. The same information was repeated in a subsequent revision of the Oriental and Australasian Ceratocanthinae (Paulian, 1978). Subsequently Cassis and Weir (1992, 2002) listed the three known species, Grebennikov et al. (2002) described the larva and pupa of *Cyphopisthes descarpentriesi* and Hawkeswood (2006) summarized published data on the subfamily, mainly under the biological point of view. Generic records of unidentified Ceratocanthinae are also reported in ecological studies on canopy beetles from two sites within the rainforests of the Australian Wet Tropics published by Stork and Grimbacher (2006) and Grimbacher and Stork (2007). I was unable however to examine the three specimens quoted in the two aforementioned papers.

Re-evaluation of Paulian’s type series of *Cyphopisthes descarpentriesi* revealed the presence of two new species among the paratypes, and examination of further material from the major Australian collections yielded two more species of *Pterorthochaetes*. The aim of this paper is therefore to provide an updated revision of the Australian Ceratocanthinae and to describe the aforementioned four new species.

The Australian Ceratocanthinae do not display a great diversity: the faunal composition falls within the Indo-Malayan element (*sensu* Matthews 2000), with endemism only at species level, suggesting a recent colonization of Australia from Asia via New Guinea. Most species occur in the rainforests of Cape York Peninsula and of the Queensland Wet Tropics, as defined by Adam (1992), however there are two species, *Cyphopisthes descarpentriesi* and *Cyphopisthes monteithi* sp. n., occurring in a completely different and drier habitat, i.e. open eucalypt woodlands and softwood and brigalow scrubs and this is very unusual, since all other Oriental and Australasian Ceratocanthinae seem to occur only in rainforests.

As a final remark it must be stressed that this revision is based on the examination of less than 50 specimens, all that the author was able to gather from museums and university collections. This shortage of available material demonstrates that a great deal of further research needs to be done in order to have a more satisfactory view of the distribution and diversity of these elusive beetles in Australia.

## Methods and acronyms

I refer to Ballerio et al. (2011) and references quoted therein for methods and terminological conventions. In the same paper a definition of the most common types of punctation was provided. Here some more remarks on the two most common types are added. Horseshoe-shaped punctation: in most cases the branches of the horseshoe are more or less parallel, however sometimes the branches tend to be convergent, so that the opening of the horseshoe is small. In these cases the horseshoe looks more or less like an ocellate puncture, but with an opening. Comma-shaped punctation: sometimes resembling commas, sometimes short horseshoes, i.e. a horseshoe with short branches, more or less like a parenthesis.

Label data are provided verbatim only for holotypes, with a slash to separate labels. In giving collecting data the author’s comments are in square brackets, while depository collection acronyms (and the accession number, when available) are in parenthesis.

Habitus photographs were taken with a Canon EOS D5 MII with a macro lens MP 65 mm, while genitalia photos were taken with a Mitutoyo M Plan APO 10x microscope objective on bellows. Serial photos were then combined with the Zerene Stacker software and cleaned and unmasked using photo processing software.

### Abbreviations

EL maximum elytral length

EW maximum total elytral width

FIT flight intercept trap

HL maximum head length

HW maximum head width

L length

PL maximum pronotal length at middle

PW maximum pronotal width at middle

W width

ABCB Alberto Ballerio Collection, Brescia, Italy.

ANIC Australian National Insect Collection (CSIRO), Canberra, Australia

MNHN Muséum National d’Histoire Naturelle Collection, Paris, France

QM Queensland Museum Collection, Brisbane, Australia (includes also the University of Queensland Insect Collection).

QPIM Queensland Department of Primary Industries Collection, Mareeba, Australia

RMNH Naturalis Collection, Leiden, The Netherlands

## Systematics

### Genus *Cyphopisthes* Gestro, 1899

The genus is in need of a revision and, after the re-definition made by Ballerio (2000), is currently comprised of a dozen morphologically very close species (with the sole exception of *Cyphopisthes inexpectatus* Paulian, 1981, which is actually a member of the “*Perignamptus* genus group”, as defined by Ballerio 2009), ranging from India to New Guinea and Queensland (the record from New Caledonia by Paulian (1991) is doubtful). Members of the genus *Cyphopisthes* are rainforest dwellers (some Australian species are an exception), often found in termite nests or by sifting leaf litter.

Diagnosis of Australian species only: 4.5–5.0 mm in length. Reddish-brown to dark brown. Volant. Enrollment coaptations perfect, with all parts matching perfectly. Dorsum glabrous (setation is not visible at 30× magnification). Base of scutellum without a smooth raised transverse area. Elytra somewhat flattened dorsally and forming a distinct pseudepipleuron laterally. Antennae 10-segmented, with strongly securiform scape. Labrum subtruncate. Mandibles with long pointed apicalis. Head with a large dorsal ocular area, genal canthus complete (fused with or almost reaching occipital area). Protibiae sexually dimorphic (female with two outer apical teeth, male with only one outer apical tooth). Mesotibiae: apical spur of males curved inwards, in females apical spur is straight. Metatibiae with two straight apical spurs. Aedeagus with parameres weakly sclerotized, short, dorsally flattened and almost symmetrical (species specific differences usually not appreciable). Genital segment with a long manubrium (longer than or almost as long as basal triangle).

#### Cyphopisthes descarpentriesi

Paulian, 1977

http://species-id.net/wiki/Cyphopisthes_descarpentriesi

Figs 1A–D , 5B , 6

Cyphopisthes descarpentriesi Paulian, 1977: 263 (description, distribution, biology); Paulian 1978 (key, distribution); Cassis and Weir 1992 (catalogue); Grebennikov et al. 2002 (description of larva and pupa); Grebennikov et al. 2004 (key to larva); Hawkeswood 2006 (summary of published data); Ocampo and Ballerio 2006 (cheklist).

##### Material examined.

Holotype, sex undetermined, (ANIC) [enrolled specimen, glued on a point]: Queensland, 19.40S, 146.51E, Lansdown Station, Woodstock, 3 July 1974, #54, J.A.L. Watson in gallery of *Mastotermes* nest / Holotype / Holotype / *Cyphopisthes descarpentriesi* sp. n. R. Paulian det. / ANIC Database no. 25 062056. Examined paratypes: 2 exx., Lansdown Station, via Woodstock, 10 July 1979, R. A. Barrett, with *Mastotermes darwiniensis* (ANIC); 2 exx., Queensland, Pallarenda. Townsville, 1.VII.1974, J. A. L. Watson, in galleries of *Mastotermes* (MNHN, ANIC).

##### Description.

Size: HL = 0.98 mm; HW = 1.54 mm; PL = 1.63 mm; PW = 2.63 mm; EL = 2.87 mm; EW = 2.63 mm. Overall morphology as in generic description. Reddish-brown, shiny, glabrous (very fine short yellowish setation visible at 50x magnification), sternum, tarsi and antennae reddish-brown.

Head: interocular distance about six times maximum width of dorsal ocular area, punctation dense and impressed, disc with very short transverse comma shaped punctures, each one having a simple small puncture at its interior side, sides of disc with large comma-shaped punctures centrifugally oriented, with opening facing internally, each one having a simple fine puncture internally, anterior portion of clypeus with three to four irregular anastomosing transverse lines.

Pronotum: margin completely bordered, anterior angles angulate, completely covered by large, almost closed, horseshoe-shaped punctures, on disc with a small opening directed anteriad, at sides punctures larger than on disc, with a small opening directed laterad, each puncture having inside a small setigerous pore. Punctation dense: interpunctural distance being less than puncture diameter.

Scutellum: covered by dense horseshoe-shaped punctures with posterior openings.

Elytra: W/L: 0,93. Humeral callus indistinct, two short longitudinal lines starting at humerus and occupying proximal third, sutural interstria indistinct, completely and uniformly covered by impressed large horseshoe-shaped punctures with a small posterior openings, each one bearing a setigerous pore in the middle. Pseudepipleura with longitudinally oriented anastomosing horseshoe-shaped punctures mixed with comma-shaped punctures.

**Figure 1.**
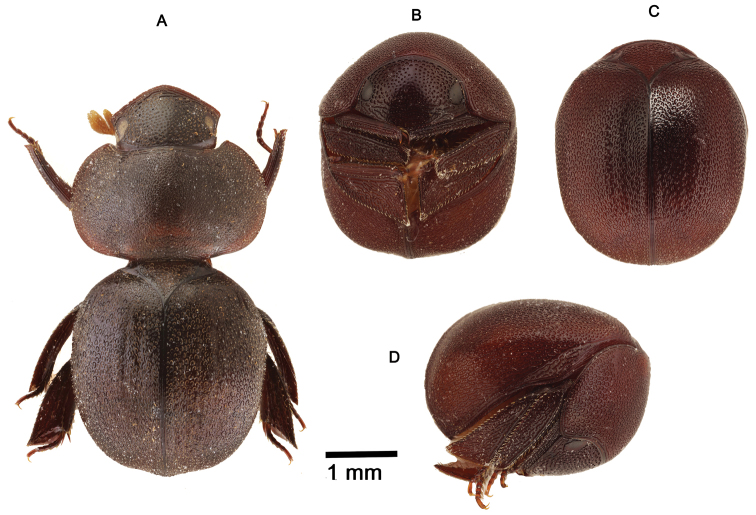
*Cyphopisthes descarpentriesi* Paulian, 1977. **A** Extended Paratype, dorsal view **B** enrolled Paratype, ventral view **C** enrolled Paratype, dorsal view **D** enrolled Paratype, lateral view.

##### Diagnosis.

*Cyphopisthes descarpentriesi* is unique among all other known Australian *Cyphopisthes* in having the pronotum and the elytra completely covered by such large and dense uniform horseshoe-shaped punctures with small posterior openings. All other known members of the genus have remarkably finer punctation and elytral punctation differs from the pronotal one.

##### Etymology.

Dedicated to André Descarpentries (1919–1998) of MNHN.

##### Distribution and habitat.

Known from north eastern Queensland coast. All specimens have been collected in open eucalypt woodland, in nests of *Mastotermes* (Isoptera). Termitophily has already been reported for *Cyphopisthes* (Ballerio and Maruyama 2010). In the same nests larvae and pupae were collected, subsequently described by Grebennikov et al. (2002). Open eucalypt woodland is a very unusual habitat for a *Cyphopisthes*, since most species are rainforest dwellers.

##### Remarks.

The type series contained three different species (two of them new to science and described below). Among paratypes of true *Cyphopisthes descarpentriesi* in ANIC one specimen had the genal canthus shortened, leaving a distinct gap between its tip and the occipital area.

Paulian (1978) identified as *Cyphopisthes descarpentriesi* also an old specimen in RMNH, ex coll. Pascoe, labelled “Mus. Godeffroy, Peak Down, Austr./10791/*Synarmostes acromialis* Pascoe”. Probably the correct locality name should be “Peak Downs” (22°15'S, 148°11'E) in Central Queensland (Federica Turco, pers. comm.). I examined two photographs of that specimen, kindly provided by Hans Huijbregts (RMNH): it is certainly not a *Cyphopisthes descarpentriesi*, and probably represents a specimen of *Cyphopisthes monteithi* sp. n. or another species very close to it.

#### Cyphopisthes monteithi sp. n.

http://zoobank.org/41532D13-A9FE-45DA-BB27-20A403215A64

http://species-id.net/wiki/Cyphopisthes_monteithi

**Figure 2.**
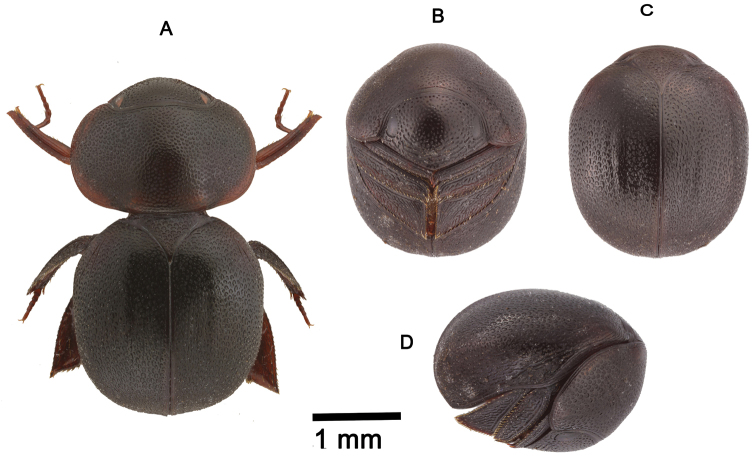
*Cyphopisthes monteithi* sp. n. **A** Extended Holotype, dorsal view **B** enrolled Holotype, ventral view **C** enrolled Holotype, dorsal view **D** enrolled Holotype, lateral view.

**Figure 3.**
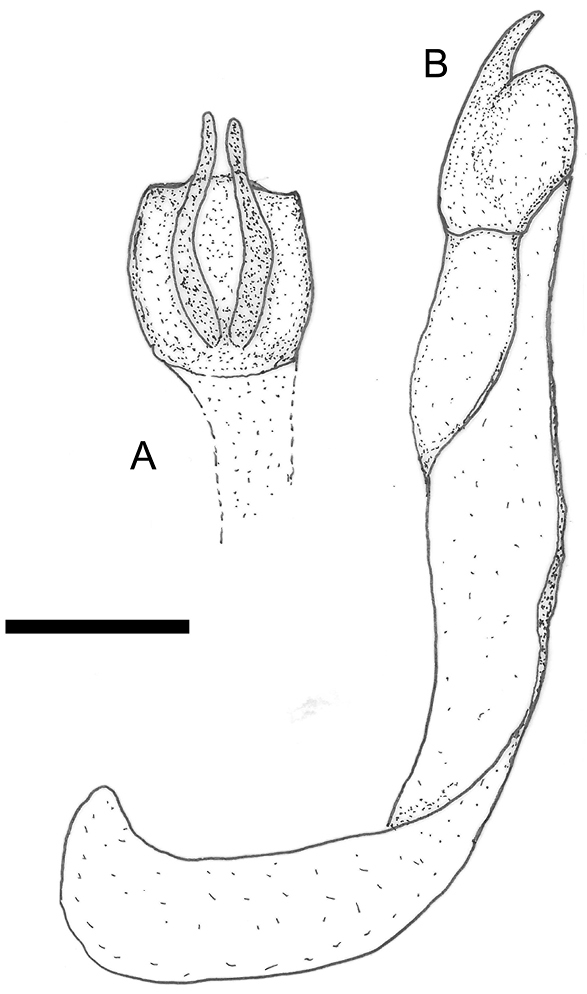
*Cyphopisthes monteithi* sp. n., aedeagus **A** dorsal view of parameres **B** lateral view of aedeagus. Scale bar: 0,1 mm.

Figs 2A–D , 3A–B , 5C , 6 , 11C

##### Type locality.

Amphitheatre scrub, Expedition Range National Park, Queensland, Australia.

##### Type material.

Holotype, male (QM, accession number: T189552) [extended specimen, glued on a card; genitalia mounted in DMHF resin on a separate card under the beetle]: Queensland, 25.13S, 148.59E, Expedition Range NP, 5063, Amphitheatre scrub, 520 m, 25 Sep–17 Dec 1997, Cook & Monteith, Vine for. Intercept; Paratype: 1 male, QLD, 24.48S, 149.45E, Brigalow Res. Stn., site 5, 16 Dec. 2000–28 Mar. 2001, D Cook & G Monteith, FIT softwood scrub 10020 (QM, accession number: T189553).

##### Further material examined

**(excluded from the type series).** 1 male: Mt. Coot-tha, Brisbane, Queensland, 13–20.III.1971, G. B. Monteith, ex leaf litter (QM) [included in the type series of *Cyphopisthes descarpentriesi* by Paulian]. 1 female, QLD, 27.58S, 152.39E, Kalbar 3 km SE, 120m, 2 Dec 2000–7 May 2001, C.J. Burwell, 10161, Brigalow scrub FIT (QM).

##### Description.

Size: HL = 0.84 mm; HW = 1.11 mm; PL = 1.22 mm; PW = 2.02 mm; EL = 2.36 mm; EW = 2.11 mm. Overall morphology as in generic description. Dark brown, shiny, glabrous (very fine short yellowish setation visible at 90×), sternum, tarsi and antennae reddish-brown.

Head: interocular distance about five times maximum width of dorsal ocular area, punctation relatively dense and impressed, disc with some impressed short transverse comma-shaped punctures, each one having a simple small puncture at its interior side, sides of disc with large comma-shaped punctures centrifugally oriented, with opening facing internally, each one having a simple fine puncture internally, anterior portion of clypeus with three-four irregular anastomosing transverse lines.

Pronotum: margin completely bordered, anterior angles angulate, completely covered by medium sized horseshoe-shaped punctures, on disc with an opening directed anteriad, punctures at sides larger than on disc, with an opening directed laterad, each puncture having inside it a small setigerous pore. Punctation dense: interpunctural distance being less than their diameter.

Scutellum: covered by dense horseshoe-shaped punctures with posterior openings.

Elytra: W/L: 0,93). Humeral callus indistinct, two short longitudinal lines starting at humerus and occupying proximal third, sutural interstria indistinct, disc with longitudinally oriented comma-shaped punctures, each one having a simple small puncture at its left side, sides of disc with medium horseshoe-shaped punctures with small posterior openings, each one bearing a setigerous pore in the middle. Interpunctural distance subequal to puncture width. Pseudepileura with longitudinally oriented anastomosing horseshoe-shaped punctures mixed with comma-shaped punctures.

Genital segment: Fig. 11C. Aedeagus: Fig. 3A, B.

##### Dignosis.

Very close to *Cyphopisthes yorkensis* sp. n., but can be easily distinguished from it by the presence of denser and larger punctation on head disc.

##### Etymology.

Noun in the genitive case. Dedicated to Dr. Geoff Monteith, former curator at Queensland Museum.

##### Distribution and habitat.

Known from southern Queensland, where all specimens have been found in dry rainforest type of vegetation, i.e. softwood and brigalow scrub, in flight intercept traps (one specimen excluded from type series was collected by leaf litter sifting).

##### Remarks.

This new species and *Cyphopisthes descarpentriesi* are the most remarkable species among the Australian Ceratocanthinae since they occur south of the Queensland Wet Tropics, in environments completely different from the environment where most other *Cyphopisthes* occur, i.e. rainforests. The vegetation type in the areas where *Cyphopisthes monteithi* has been collected is characterized by drier, lower and sparser woodland, with patches off denser forest (“dry rainforest”).

I excluded from the type series the specimen from Mount Coot-tha which differs from the holotype because of the more extended comma-shaped punctation on the disc and the presence of transverse lines in the pseudepipleura and the specimen from Kalbar, which has much sparser punctation on the head. This circumstance, together with the fact that they occur some 480 km Southeast of the type locality of *Cyphopisthes monteithi*, suggests prudence before assigning them to the new species. The same applies to the above mentioned specimen from Peak Downs (see under *Cyphopisthes descarpentriesi*).

#### Cyphopisthes yorkensis sp. n.

http://zoobank.org/9046ACE0-1DCA-4D1C-B7AF-DA70F9949875

http://species-id.net/wiki/Cyphopisthes_yorkensis

Figs 4A–D , 5A , 6

##### Type locality.

Iron Range, Cape York Peninsula, Queensland, Australia.

##### Type material.

Holotype, female (QM, accession number: T189554): North Queensland, Iron Range, Cape York Pen., 1–9 June 1971, G. B. Monteith [extended specimen, glued on a card]. Paratypes: 1 ex., sex undetermined, same data as holotype (MNHN); 1 ex., sex undetermined: 12.44S, 143.14E, 3km ENE of Mt. Tozer, QLD, 28 Jun–4 Jul. 1986, T. Weir & A. Calder (ANIC).

##### Description.

Size: HL = 0.80 mm; HW = 1.28 mm; PL = 1.24 mm; PW = 2.22 mm; EL = 2.42 mm; EW = 2.33 mm. Overall morphology as in generic description. Dark reddish-brown, shiny, glabrous (very fine short yellowish setation visible at 50×), sternum, tarsi and antennae reddish-brown.

Head: four to five anastomosing irregular transverse lines at anterior portion of clypeus, clypeal disc almost smooth, with only a few sparse fine simple punctures, sides of disc and frons with denser, bigger short comma-shaped punctures. Interocular distance about seven times the maximum width of dorsal ocular area.

Pronotum: margin completely bordered, anterior margin thicker than lateral and basal margin, anterior angles angulate. Punctation: on disc small horseshoe-shaped punctures with an opening directed anteriad, each one containing a fine simple puncture in middle, at sides punctures larger (about twice the size of discal punctures) than on disc, with an opening directed laterad, each puncture having inside it a small setigerous pore. Base with smaller comma-shaped punctures with openings directed anteriad. Punctation dense: interpunctural distance being shorter than, to equal to, puncture diameter.

Scutellum: covered by dense horseshoe-shaped punctures with posterior openings.

Elytra: (W/L: 0,93). Humeral callus indistinct, two short longitudinal lines (the inner being slightly shorter than the outer) starting at humerus and occupying the proximal third, sutural interstria indistinct, disc with longitudinally oriented comma-shaped punctures, each one having a simple small puncture at its internal side, humeral punctation made of short transverse comma-shaped punctures becoming horseshoe-shaped towards disc, with posterior openings, sides of elytral dorsum with longitudinally oriented long comma-shaped punctures opening laterad and a simple fine puncture at their outer side, punctation dense: interpunctural distance being shorter than their width. Pseudepileura with longitudinally oriented comma-shaped punctures.

**Figure 4.**
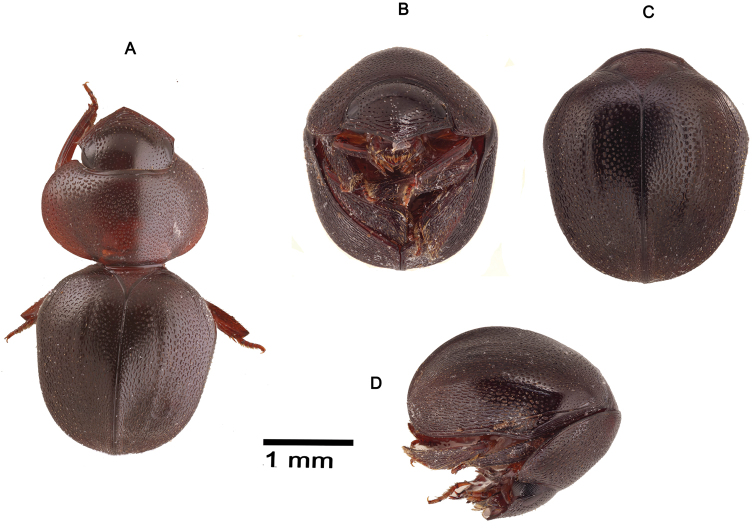
*Cyphopisthes yorkensis* sp. n. **A** Extended Holotype, dorsal view **B** enrolled Holotype, ventral view **C** enrolled Holotype, dorsal view **D** enrolled Holotype, lateral view.

**Figure 5.**
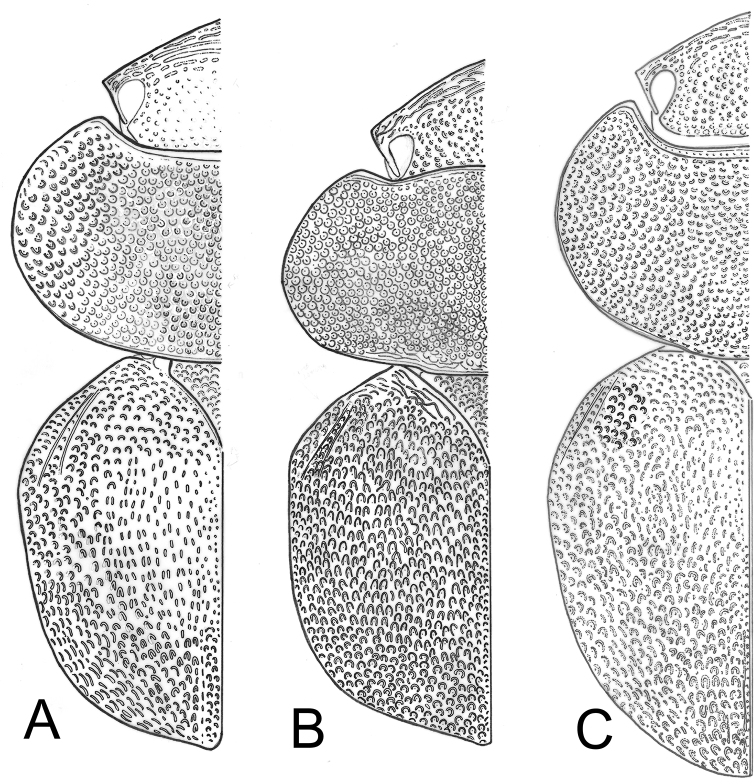
Outline of punctation pattern in **A** *Cyphopisthes yorkensis* sp. n. **B** *Cyphopisthes descarpentriesi* Paulian, 1977 **C** *Cyphopisthes monteithi* sp. n. (drawings by Mario Toledo).

**Figure 6.**
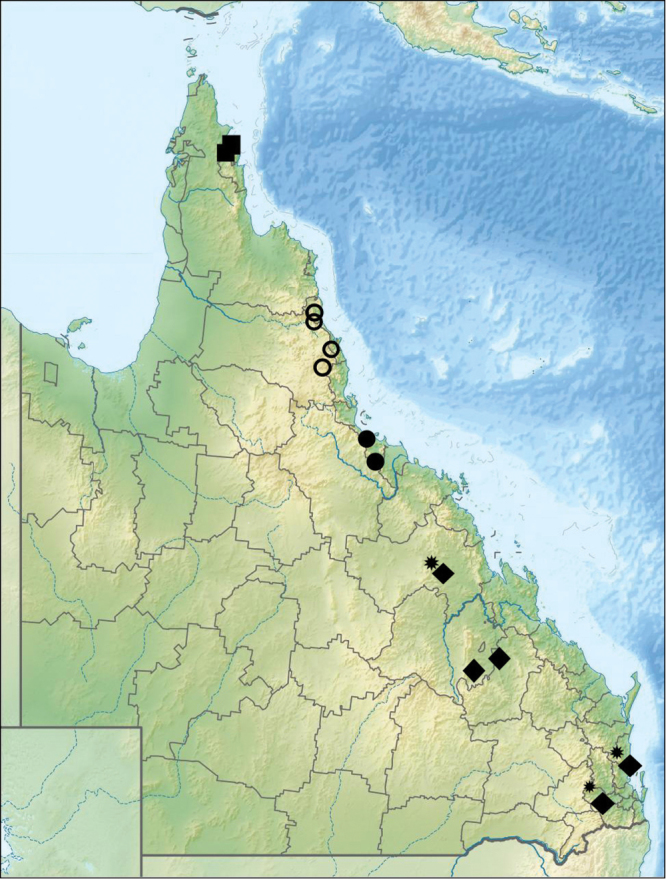
map of Queensland showing distribution of *Pterorthochaetes danielsi* sp. n., *Pterorthochaetes cribricollis* Gestro, 1899 and *Cyphopisthes yorkensis* sp. n. (black squares); *Pterorthochaetes storeyi* sp. n. (empty circles); *Cyphopisthes descarpentriesi* Paulian, 1977 (black circles); *Cyphopishtes monteithi* sp. n. (black rhombuses); *Cyphopisthes* cf. *monteithi* (black rhombuses with asterisk).

##### Diagnosis.

Very close to the New Guinean *Cyphopisthes amphicyllis* (Sharp, 1875), because of the sparse fine punctation on the disc of head. The new species differs from it because of the more impressed punctation of the elytra, which is also slightly sparser than in *Cyphopisthes amphicyllis* and has many more comma-shaped punctures, whereas in *Cyphopisthes amphicyllis* the dominant type of punctation is horseshoe-shaped. Among Australian species it can be easily distinguished because of the sparse and fine punctation of clypeal disc and the more extended comma-shaped punctation on elytral disc.

##### Etymology.

Latin adjective in the nominative singular, meaning “from York”. Named after the type locality.

##### Distribution and habitat.

Known from the Cape York Peninsula only (northern Queensland), where it occurs in the lowland rainforests of Iron Range and Mount Tozer.

##### Remarks.

Holotype and the paratype in MNHN were part of the type series of *Cyphopisthes descarpentriesi*.

### Genus *Pterorthochaetes* Gestro, 1899

About 25 species are ascribed to the genus, but a revision in progress will probably more than double the number of species (Ballerio, in prep.). The distribution ranges from India and Sri Lanka to Vanuatu. Members of the genus *Pterorthochaetes* are rainforest dwellers, often found under the bark of dead logs, sometimes in association with Passalidae (Kon et al. 2010, Ballerio and Maruyama 2010), by sifting leaf litter or in termite nests. The morphology is relatively uniform and the most useful characters for species recognition are found in the male genitalia (shape of parameres and sclerotisations of the internal sac) and female genitalia (bursal sclerites) (see Ballerio 1999).

Diagnosis for Australian species only: 6 to 8 mm in length. Dark brown to black. Volant. Enrollment coaptations perfect, with all parts matching perfectly. Dorsum setose (setae short and thick). Elytra regularly convex, without any distinct pseudepipleuron. Base of scutellum with a smooth raised transverse area (very reduced in *Pterorthochaetes cribricollis*). Antennae 9-segmented, scape clavate. Mandibles with short pointed apices. Labrum not truncate, somewhat depressed distally. Head with a medium sized ocular area, genal canthus almost complete (not fused with the occipital area). Mesotibiae: with only one apical spur, in males the inner apical mesotibial angle is acutely expanded (false spur). Male metatibiae with one twisted apical spur and one straight apical spur. Aedeagus with parameres fairly sclerotized and asymmetrical. Genital segment with short manubrium. Female genitalia with bursa copulatrix with two paired symmetrical/asymmetrical sclerites (bursal sclerites).

#### Pterorthochaetes cribricollis

Gestro, 1899

http://species-id.net/wiki/Pterorthochaetes_cribricollis

**Figure 7.**
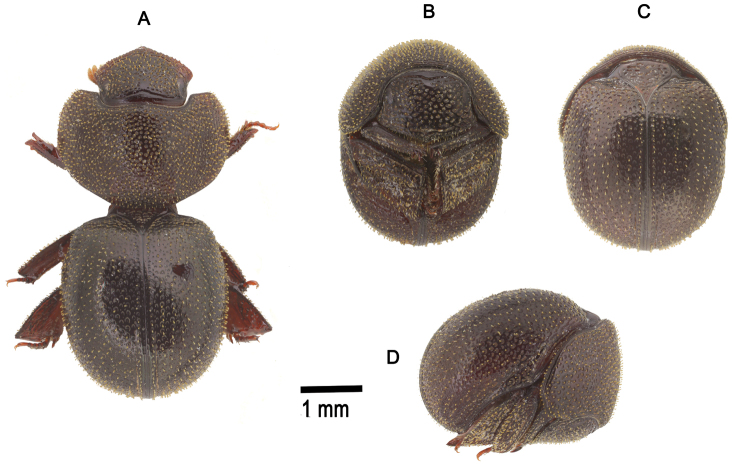
*Pterorthochaetes cribricollis* Gestro, 1899 **A** Extended specimen, dorsal view **B** enrolled specimen, ventral view **C** enrolled specimen, dorsal view **D** enrolled specimen, lateral view.

Figs 6 , 7A–D , 11D , 12A , 13A–C , 14C

Pterorthochaetes cribricollis Gestro, 1899: 37 (description, distribution, key); Paulian 1978 (key, distribution); Cassis and Weir 1992 (catalogue); Ocampo and Ballerio 2006 (checklist)

##### Material examined.

10 specimens [two males and two females dissected]: 4 males and 2 females, Iron Range, Cape York Pen., N. Qld. 28 Apr.–5 May 1968. G. Monteith (QM); 1 female, Iron Range, Cape York Pen., N. Qld. 11–17 May 1968. G. Monteith (QM); 1 female, Iron Range, Cape York Pen., N. Qld. 26 May–2 June 1971 B. K. Cantrell (QM); 1 female, QLD: 12.710°S, 143.291°E, Cooks Hut, Iron Range, 5 m, 15 Dec 2010, Monteith, Escalona & Will, hand and at HV light 34817 (QM).

##### Description.

Size: HL = 0.90 mm; HW = 1.30 mm; PL = 1.32 mm; PW = 2.20 mm; EL = 2.25 mm; EW = 2.15 mm. Overall morphology as in generic description. Dark brown, shiny, setation yellowish, sternum, tarsi and antennae reddish-brown.

Head: completely and uniformly covered by impressed comma-shaped punctures with posterior openings, spaced out by a distance of about half their diameter. Anterior portion of clypeus with one or two irregular transverse anastomosing lines. Interocular distance about 9 times the maximum width of dorsal ocular area.

Pronotum: margins completely bordered, lateral margins with a row of erect thick yellowish slightly clavate setae, about as long as the distance between them. Pronotal setation made of thick medium sized clavate yellowish setae, punctation as follows: disc covered by impressed short transverse comma-shaped punctures, with posterior openings and containing a small fine setigerous pore, sides with a few larger more curved comma-shaped punctures opening backwards.

Scutellum: basally with two longitudinal irregular rows of horseshoe-shaped punctures, uniting towards apex.

Elytra: humeral callus poorly pronounced, sutural stria occupying medial and distal third. Elytral punctation as follows: uniformly covered by irregular longitudinal rows of mixed simple impressed small punctures and medium-sized horseshoe-shaped punctures opening backwards, larger on sides and apical third. Interpunctural distance subequal to puncturelength.

Aedeagus: basal piece about three times as long as parameres. Parameres slightly asymmetrical, internal sac distally with some irregular weak sclerotisations (Fig. 12A, Fig. 13A–C).

Male genital segment: as in Fig. 11D.

Bursal sclerites: slightly asymmetrical, as in Fig. 14C.

##### Diagnosis.

Easily distinguished from all other Australian *Pterorthochaetes* because of the combination of smaller size, the pattern of punctation of pronotum and elytra, which on pronotum is only made of short impressed transverse comma-shaped punctures, whereas all other Australian species have, at least partly, horse-shoe shaped punctures, often with a very small posterior openings, while on elytra is made of a much smaller punctation compared to *Pterorthochaetes danielsi* sp. n. and much denser compared to *Pterorthochaeres storeyi* sp. n. and *Pterorthochaetes simplex* Gestro, 1899.

##### Etymology.

From Latin *cribratus* (profusely perforated) and *collis* (pronotum), due to the dense and impressed punctation.

##### Distribution and habitat.

In Australia known from the lowland rainforests of Iron Range (Northern Queensland). This species occurs also in New Guinea (type locality: Papua New Guinea, Central Province, lower Kemp Welch River, Ighibirei).

##### Remaks.

identification was made by comparison with the holotype from New Guinea in Museo Civico di Storia Naturale “G. Doria”, Genova.

#### Pterorthochaetes danielsi sp. n.

**Figure 8.**
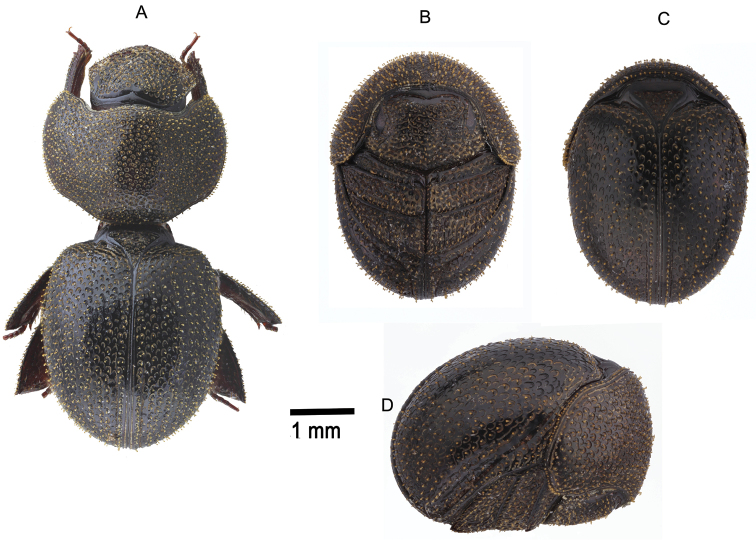
*Pterorthochaetes danielsi* sp. n. **A** Extended Holotype dorsal view **B** enrolled Paratype, ventral view **C** enrolled Paratype, dorsal view **D** enrolled Paratype, lateral view.

http://zoobank.org/D3EAF3F7-6DE8-4093-9ABB-8E8D9F69327D

http://species-id.net/wiki/Pterorthochaetes_danielsi

Figs 6 , 8A–D , 11B , 12C , 13D–F , 14D

##### Type locality.

West Claudie River, Iron Range, Queensland, Australia.

##### Type material.

Holotype, male (QM, accession number: T189544): Australia: Queensland: NE: West Claudie R., Iron Range, 3 Dec. 1985, G. Monteith / QM Berlesate no. 690 12.45S, 143.14E Rainanteriorst 50m Stick brushing. [extended specimen, glued on a card, dissected, genitalia mounted in DMHF resin on a separate card, same pin]. Allotype: 1 female [dissected], Iron Range, Cape York Pen., N. Qld. 28 Apr.–5 May 1968. G. Monteith (QM, accession number: T189548). Paratypes [all dissected]: 1 male, same data as holotype (ABCB); 1 male, West Claudie R., Iron Range, N. Qld., 3–10 Dec. 1985, G. Monteith & D. Cook, Pyrethrum knockdown/RF (QM, accession number: T189551); 1 male, Iron Range, Cape York Pen., N. Qld. 5–10 May 1968. G. Monteith (MNHN); 2 males, Iron Range, Cape York Pen., N. Qld. 28 Apr.–5 May 1968. G. Monteith (QM, accession numbers: T189555 and T189556); 1 female, QLD:12.714°S, 143.287°E, East Claudie River, 15 m, 9 Dec 2010 34778, G. Monteith, Bark spray (QM, accession number: T189773).

##### Description.

HL = 0.75 mm; HW = 1.60 mm; PL = 1.75 mm; PW = 2.55 mm; EL = 3.00 mm; EW = 2.60 mm. Overall morphology as in generic description. Dark brown, shiny, setation yellowish, sternum, tarsi and antennae reddish-brown.

Head: completely and uniformly covered by impressed coarse horseshoe-shaped punctures, anastomosing on disc. Anterior portion of clypeus with irregular transverse anastomosing lines. Interocular distance about 11 times maximum width of dorsal ocular area.

Pronotum: margins completely bordered, lateral margins with a row of erect thick yellowish simple setae, about as long as the distance between them. Pronotal setation made of thick medium sized clavate yellowish setae, punctation as follows: disc covered by impressed transverse small horseshoe-shaped punctures, with posterior openings and containing a small fine setigerous pore, sides of disc with a few large ocellate punctures and sides of pronotum with larger horseshoe-shaped punctures with opening laterad. Anterior angles having six longitudinal irregular lines. Distance between punctures distinctly less than their diameter.

Scutellum: basally with two longitudinal irregular rows of horseshoe-shaped punctures, uniting towards apex.

Elytra: humeral callus poorly pronounced, sutural stria occupying the medial and distal third. Elytral punctation as follows: uniformly covered by large horseshoe-shaped punctures, some punctures becoming ocellate at apical third and at sides of elytra. Each horseshoe-shaped and ocellate puncture enclosing a small fine simple puncture bearing a clavate yellowish seta. Interpunctural distance on elytra distinctly less than puncture diameter.

Aedeagus: basal piece about twice length of parameres. Parameres slightly asymmetrical, internal sac distally with some irregular weak sclerotisations (Fig. 13D–F, Fig. 12C).

Male genital segment: as in Fig. 11B.

Bursal sclerites: slightly asymmetrical, as in Fig. 14D, subject to some variability.

##### Diagnosis.

Very close to the New Guinean *Pterorthochaetes brevis* (Sharp, 1875), from which differs mainly by the punctation of pronotal disc, which in *Pterorthochaetes brevis* is sparser and of elytra, which in *Pterorthochaetes brevis* is shallower and sparser. Among the other Australian species it can be easily distinguished by having the elytral punctation in the form of dense, large horseshoe-shaped punctures, almost without isolated simple punctures. The shape of the bursal sclerites, while very similar to those of *Pterorthochaetes brevis*, is unique within the Australian *Pterorthochaetes*.

##### Etymology.

Noun in the genitive case. Dedicated to Gregory Daniels, former collections manager at University of Queensland Insect Collection, Brisbane.

##### Distribution and habitat.

Known from the Cape York Peninsula only (Northern Queensland), where it occurs in the lowland rainforests of Iron Range.

#### Pterorthochaetes simplex

Gestro, 1899

http://species-id.net/wiki/Pterorthochaetes_simplex

Figs 6 , 9A–B , 14B

Pterorthochaetes simplex : Gestro, 1899: 36 (description, distribution, key); Paulian 1978 (key, distribution); Cassis and Weir 1992 (catalogue); Ocampo and Ballerio 2006 (checklist)

##### Material examined.

Holotype, female (MNHN): Australie, Queensland / Typus / dr. Gestro vidit / holotype / *Pterorthochaetes simplex*, Typus ! Gestro. [extended, glued on a card, in good condition, dissected by the present author with bursal sclerites mounted in DMHF resin on a separate card under the specimen].

##### Description.

Size: HL = 0.70 mm; HW = 1.30 mm; PL = 1.36 mm; PW = 2.30 mm; EL = 2.36 mm; EW = 2.00 mm. Overall morphology as in generic description. Dark brown, shiny, setation yellowish, sternum, tarsi and antennae reddish-brown.

Head: completely and uniformly covered by comma-shaped punctures, anastomosing on disc. Anterior portion of clypeus with three irregular transverse anastomosing lines. Interocular distance about 13 times the maximum width of dorsal ocular area.

Pronotum: margins completely bordered, lateral margins with a row of erect thick yellowish simple setae. Pronotal setation made of fine short simple yellowish setae, punctation as follows: disc covered by shallow sparse ocellate punctures, containing a small fine setigerous pore, sides with sparse shallow ocellate punctures larger than on disc mixed with a few large horseshoe-shaped punctures with small posterior openings. Anterior angles having six longitudinal irregular lines. Distance between punctures distinctly less than their diameter.

Scutellum: basally with two longitudinal irregular rows of horseshoe-shaped punctures, uniting towards apex.

Elytra: humeral callus poorly pronounced, sutural stria occupying medial and distal third. Elytral punctation as follows: uniformly covered by large shallow sparse horseshoe-shaped punctures mixed with very fine simple punctures, interpunctural distance being equal to their diameter.

Bursal sclerites: slightly asymmetrical, as in Fig. 14B.

Male unknown.

**Figure 9.**
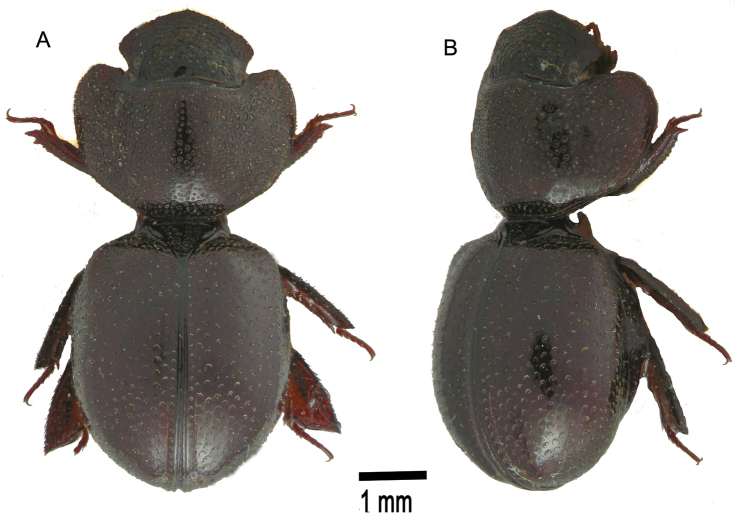
*Pterorthochaetes simplex* Gestro, 1899 **A** Extended Holotype, dorsal view **B** extended Holotype lateral view.

##### Diagnosis.

Due to the large, shallow sparse punctation this species can be easily identified among all other Australian *Pterorthochaetes*. In particular the pronotal punctation is unique, being shallow, large and almost ocellate, while the elytral punctation is sparser than in *Pterorthochaetes danielsi* sp. n. and larger and shallower than in *Pterorthochaeres storeyi* sp. n.. The shape of the bursal sclerites is also very distinctive.

##### Etymology.

Latin *simplex* (simple), probably due to the punctation of dorsum, shallower and sparser than in most other *Pterorthochaetes*.

##### Distribution and habitat.

Unknown. The holotype bears a generic label indicating “Queensland”. Paulian (1977) cites further specimens from Queensland in the museums of Canberra and Brisbane, but I was unable to locate specimens belonging to this species in the aforementioned museums. All the specimens bearing an identification label as *Pterorthochaetes simplex* by Paulian belonged to the two new species herein described or, in the case of the specimen from New Guinea listed by Paulian, to a further new species not occurring in Australia. I was unable to locate the two specimens from “Churchill Creek (16.34°S, 145.19°E)” and “Mt Lewis road, via Julatten”, both localities, however, fall within the range of *Pterorthochaeres storeyi* sp. n. The records of *Pterorthochaetes simplex* from Daintree by Grove (2000) actually refer to *Pterorthochaeres storeyi* sp. n. (see below).

##### Remarks.

As correctly stated in Paulian (1977) the holotype is kept in Paris (ex coll. Oberthür) and not in Genoa, as mistakenly reported in Paulian (1978).

#### Pterorthochaetes storeyi sp. n.

http://zoobank.org/D5822479-72B5-409C-A567-D9432241C361

http://species-id.net/wiki/Pterorthochaetes_storeyi

Fig. 6 , 10A–D , 11A , 12B , 13G, H, I , 13A, E, F

##### Type locality.

Thompson Creek, Daintree, Queensland, Australia.

##### Type material.

Holotype, male (QM, registration number QMT93436): Daintree, NE Queensland: Thompson Creek, 16°06.31S, 145°26.25E, 140 m, Trunk FIT #16, 09/11/98-19/12/98, leg. Simon Grove. [extended specimen, glued on a card, dissected, genitalia mounted in DMHF resin on a separate card, same pin]. Allotype: 1 female [dissected], 1 female, Daintree, NE QLD: Thompson Creek, 16°06.31S, 145°26.25E, 140 m, Trunk FIT #24, 19/12/98–26/01/99, leg. Simon Grove (QPIM). Paratypes [6 males and 5 females dissected]: 1 male, Daintree, NE QLD: Thompson Creek, 16°06.31S, 145°26.25E, 140 m, Trunk FIT #01, 09/11/98-19/12/98, leg. Simon Grove (ABCB); 1 female, Daintree, NE QLD: Thompson Creek, 16°06.31S, 145°26.25E, 140 m, Trunk FIT #9, 19/12/98–26/01/99, leg. Simon Grove (QPIM); 1 female, Daintree, NE QLD: Thompson Creek, 16°06.31S, 145°26.25E, 140 m, 04/02/99, Night hand colln. #E9, leg. Simon Grove (QPIM); 1 male, Australia, N. Qld., Tully Falls S. F. 730 m, 18 km SSW Ravenshoe, 18.I.1988, Storey & Dickinson (QPIM); 1 male, Australia, N. Qld., Danbulla S. F., 1 km NE of Yungaburra, 13.II–6.III.1987, Storey & De Faveri (QPIM); 1 male, Worgabel S. F. via Atherton, 26.XII.1988, R. I. Storey at light (QPIM); 1 male, NEQ: 16.26S, 145.20E, O’Donoghue’s Falls, 15–16 May 1995, 150 m, leg. Monteith, Ford & Slaney (QM, accession number T189543); 1 female, Daintree, NE QLD: Thompson Creek, 16°06.31S, 145°26.25E, 140 m, Trunk FIT #14, 09/12/98-26/01/99, leg. Simon Grove (QM, accession number: T189774); 1 male, Daintree, NE QLD: Thompson Creek, 16°06.31S, 145°26.25E, 140 m, 05/02/99, Trunk Knockdown #24, leg. Simon Grove (QM, accession number: T189775); 1 male, Daintree, NE QLD: Thompson Creek, 16°06.31S, 145°26.25E, 140 m, Trunk FIT #8, 09/12/98–26/01/99, leg. Simon Grove (QM, accession number: T189776); 1 male, QLD: 17.221°S, 145.761°E, Goldsborough Rd. 12.5 km past bridge, 16–17 Sept 2010, G. Monteith RF Barkspray 34575 (QM, accession number: T189777); 1 female, QLD: 16.202°S, 145.409°E, Lync-Haven Daintree Area, 2 Dec 2012, F. Turco, rainforest, 35 m, barkspray on logs, 18742 (ABCB).

##### Description.

Size: HL = 0.70 mm; HW = 1.44 mm; PL = 1.50 mm; PW = 2.40 mm; EL = 2.66 mm; EW = 2.29 mm. Overall morphology as in generic description. Black, shiny, setation yellowish, sternum, tarsi and antennae reddish-brown.

Head: completely and uniformly covered by impressed coarse punctation, punctures transverse, comma shaped on disc, horseshoe-shaped (with opening towards internal side) at sides of disc and on frons. Anterior portion of clypeus with irregular transverse anastomosing lines. Interocular distance about 11 times the maximum width of dorsal ocular area.

Pronotum: margins completely bordered, lateral margins with a row of erect thick yellowish simple setae, longer than their distance. Pronotal setation made of thick medium sized clavate yellowish setae. Punctation as follows: disc covered by impressed transverse comma shaped punctures, with posterior openings and having a small fine setigerous pore near inferior side, sides of disc with a few ocellate punctures and sides of pronotum with larger horseshoe-shaped punctures with opening directed laterad. Anterior angles having six longitudinal irregular lines. Distance between punctures subequal to their diameter.

Scutellum: basally with two longitudinal irregular rows of horseshoe-shaped punctures, uniting towards apex.

Elytra: humeral callus poorly pronounced, sutural stria occupying the medial and distal third. Elytral punctation as follows: mixed simple fine punctures and longitudinal comma-shaped punctures with opening laterad, becoming horseshoe-shaped at apical third and at sides of elytra. Each comma-shaped and horseshoe-shaped puncture bearing bearing a clavate yellowish seta. Apical third of elytra with a few ocellate punctures. Interpunctural distance on elytra being larger than the diameter of punctures.

Aedeagus: basal piece about two times as long as parameres. Parameres slightly asymmetrical, internal sac with distally some irregular weak sclerotisations (Fig. 12B, Fig. 13G–I).

Male genital segment: as in Fig. 11A.

Bursal sclerites: strongly asymmetrical, as in Fig. 14A, E, F and subject to strong variability.

**Figure 10.**
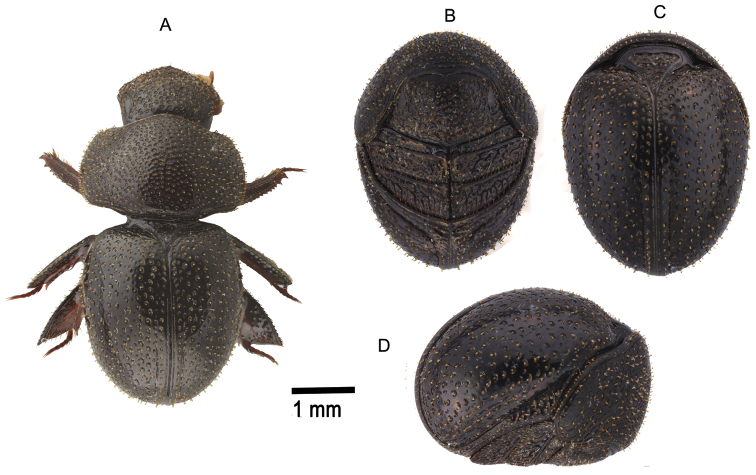
*Pterorthochaetes storeyi* sp. n. **A** Extended Paratype, dorsal view **B** enrolled Paratype, ventral view **C** enrolled Paratype, dorsal view **D** enrolled Paratype, lateral view.

**Figure 11.**
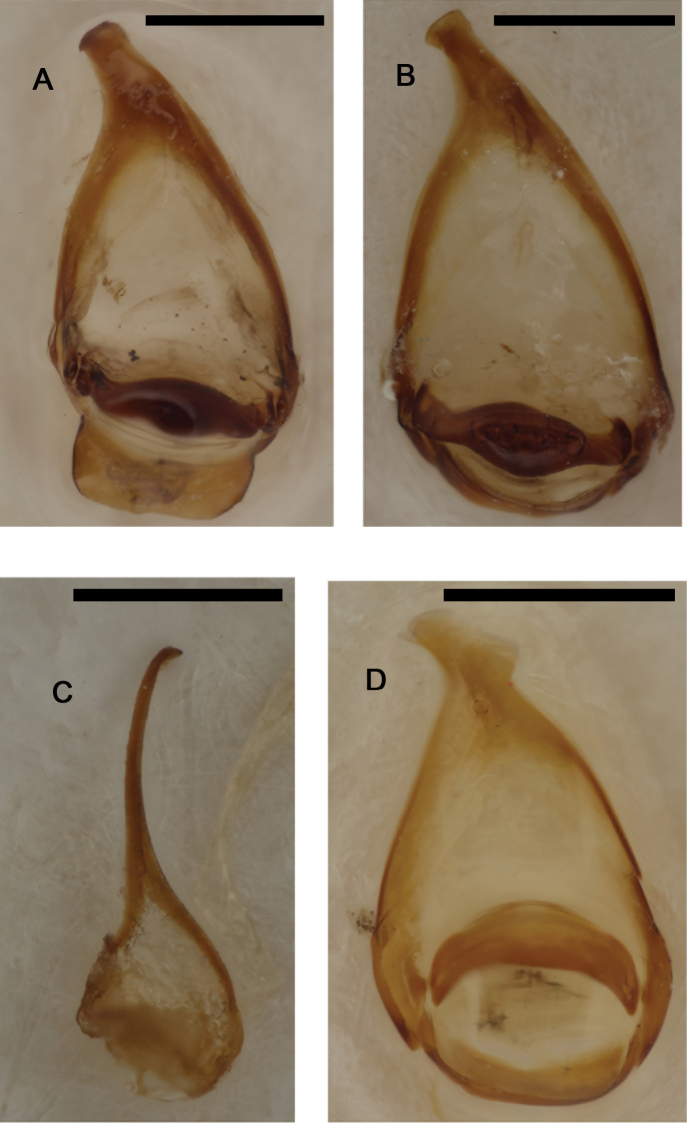
Genital segments of: **A** *Pterorthochaetes storeyi* sp. n. **B** *Pterorthochaetes danielsi* sp. n. **C** *Cyphopisthes monteithi* sp. n. **D** *Pterorthochaetes cribricollis* Gestro, 1899. Scale bar: 0,25 mm.

**Figure 12.**
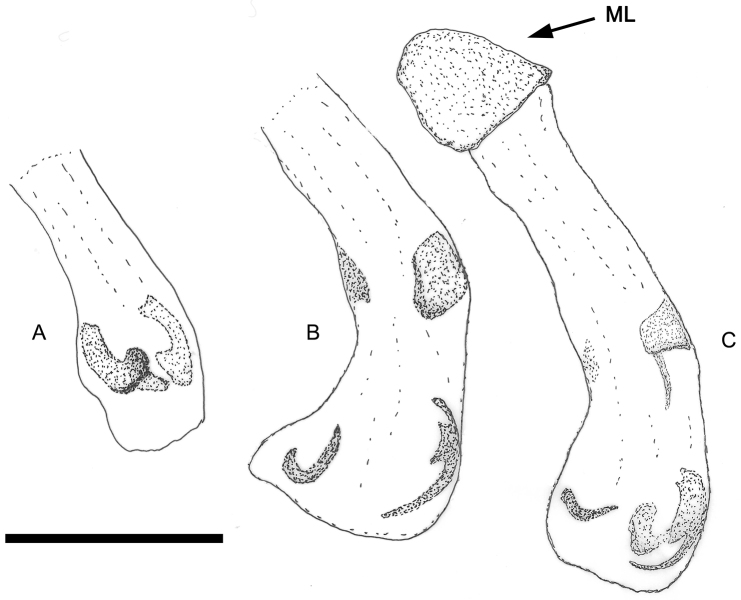
Internal sac of A *Pterorthochaetes cribricollis* Gestro, 1899 (distal portion only) **B** *Pterorthochaetes storeyi* sp. n. **C** *Pterorthochaetes danielsi* sp. n. (ML= median lobe). Scale bar: 0,5 mm.

**Figure 13.**
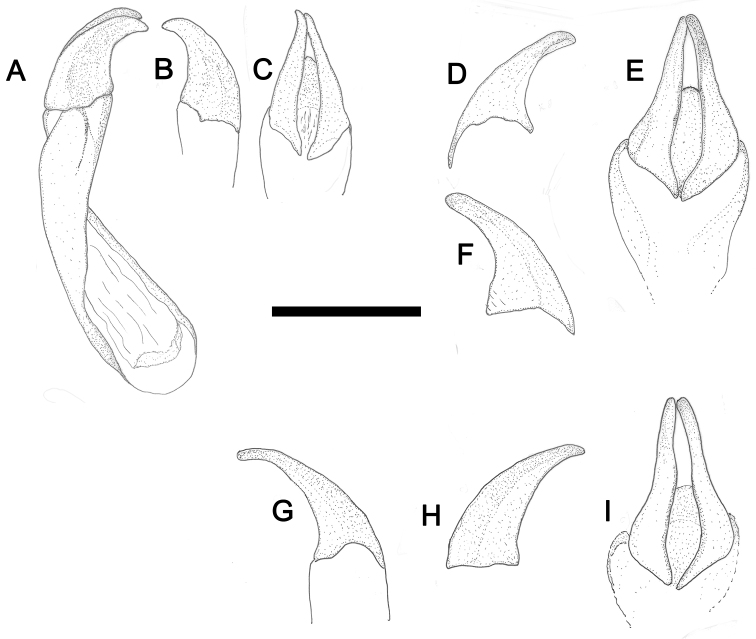
Aedeagus of: *Pterorthochaetes cribricollis* Gestro, 1899 **A** (aedeagus in lateral view) **B** (left paramere in lateral view) **C** (parameres in dorsal view); *Pterorthochaetes danielsi* sp. n. **D** (right paramere in lateral view) **E** (parameres in dorsal view) **F** (left paramere in lateral view); *Pterorthochaetes storeyi* sp. n. **G** (left paramere in lateral view) **H** (right paramere in lateral view) **I** (parameres in dorsal view). Scale bar: 0,5 mm.

**Figure 14.**
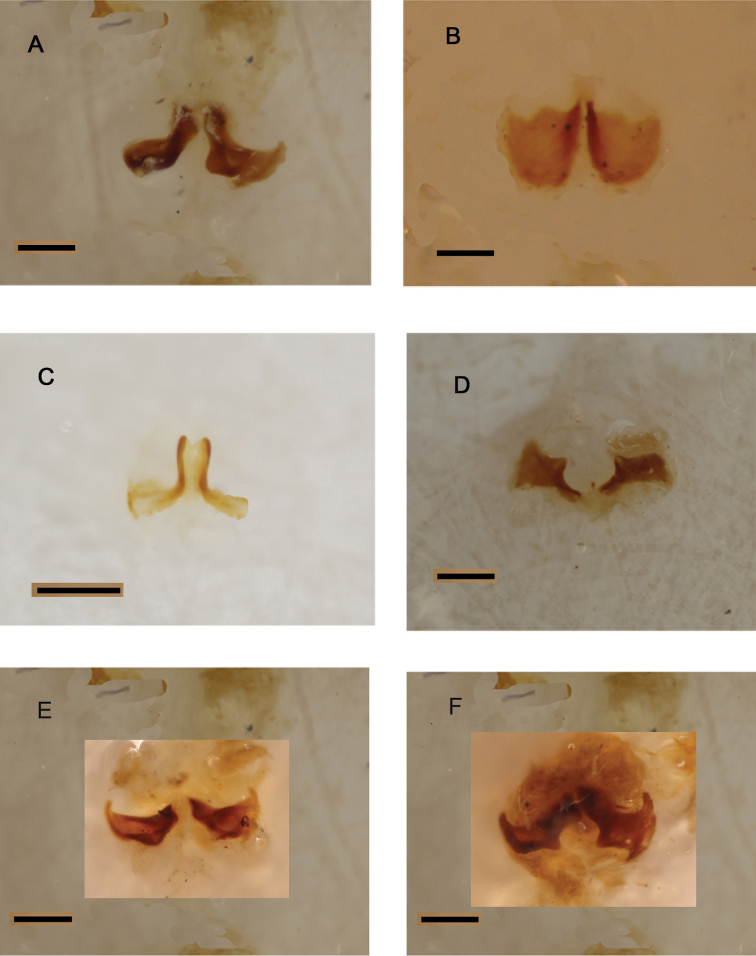
Bursal sclerites of: **A** *Pterorthochaetes storeyi* sp. n. **B** *Pterorthochaetes simplex* Gestro, 1899 **C** *Pterorthochaetes cribricollis* Gestro, 1899 **D** *Pterorthochaetes danielsi* n.sp. **E** *Pterorthochaetes storeyi* sp. n. **F** *Pterorthochaeres storeyi* sp. n.. Scale bar: 0,2 mm.

##### Diagnosis.

*Pterorthochaetes storeyi* sp. n. can be easily identified among the other Australian *Pterorthochaetes* because of the distinctive punctation pattern of elytra, with punctures sparser and shorter than in *Pterorthochaetes danielsi* sp. n. (usually with long comma-shaped punctures, rather than true horseshoe-shaped punctures as in *Pterorthochaetes danielsi*), smaller, shorter and more impressed than in *Pterorthochaetes simplex*. The shape of bursal sclerites is also very distinctive and unique within the Australian *Pterorthochaetes*.

##### Etymology.

Dedicated to Ross Storey (1949–2008), former technician at Queensland Department of Primary Industries, Mareeba. Noun in the genitive case.

##### Distribution and habitat.

Known from the Queensland Wet Tropics (sensu Adam 1992), where it occurs in lowland rainforest areas. Adults were collected mainly with flight intercept traps or at light. The *Pterorthochaetes simplex* quoted by Grove (2000) are actually specimens of *Pterorthochaeres storeyi* sp. n.

### Key to the genera and species of Australian Ceratocanthinae

**Table:** 

1	Antennae 10-segmented, labrum subtruncate, elytra with a distinct pseudepipleuron	*Cyphopisthes* Gestro, 1899, 2
–	Antennae 9-segmented, labrum not truncate, somewhat depressed distally, elytra without a distinct pseudepipleuron	*Pterorthochaetes* Gestro, 1899, 4
2	Pronotum and elytra with uniform sculpturing consisting of dense large uniform horseshoe-shaped punctures	*Cyphopisthes descarpentriesi* Paulian, 1977
–	Pronotum and elytra with smaller punctation, elytral disc mainly with longitudinal comma-shaped punctures mixed with simple punctures	3
3	Head with disc having relatively dense punctation, made of impressed very short comma-shaped punctures each one next to a very fine simple puncture	*Cyphopisthes monteithi* sp. n.
–	Head disc having very sparse punctation, made of very fine simple punctures	*Cyphopisthes yorkensis* sp. n.
4	Pronotum and elytra with transverse comma-shaped and transverse short horseshoe-shaped punctation only	*Pterorthochaetes cribricollis* Gestro, 1899
–	Pronotum and elytra with horseshoe-shaped or ocellate punctation, sometimes mixed with comma-shaped punctures	5
5	Sides of pronotum mainly with shallow large ocellate punctures	*Pterorthochaetes simplex* Gestro, 1899
–	Sides of pronotum mainly with horseshoe-shaped punctures	6
6	Pronotal disc mainly with short horseshoe-shaped punctures, medial and proximal third of elytra with dense short horseshoe-shaped punctures, only very rare simple punctures	*Pterorthochaetes danielsi* sp. n.
–	Pronotal disc mainly with transverse comma-shaped punctures,medial and proximal third of elytra with sparse short longitudinal comma-shaped punctures mixed with simple punctures	*Pterorthochaeres storeyi* sp. n.

## Supplementary Material

XML Treatment for Cyphopisthes descarpentriesi

XML Treatment for Cyphopisthes monteithi

XML Treatment for Cyphopisthes yorkensis

XML Treatment for Pterorthochaetes cribricollis

XML Treatment for Pterorthochaetes danielsi

XML Treatment for Pterorthochaetes simplex

XML Treatment for Pterorthochaetes storeyi
